# Physical exercise mitigates motor and muscular deficits in the 3xTg-AD model of Alzheimer’s disease

**DOI:** 10.3389/fnagi.2026.1730578

**Published:** 2026-01-27

**Authors:** Jesús Andrade-Guerrero, Karen León-Arcia, Omar Emiliano Aparicio-Trejo, Belen Cuevas-López, Oscar Arias-Carrión, Sofía Y. Díaz-Miranda, Luis O. Soto-Rojas

**Affiliations:** 1 Laboratorio de Investigación en Neurociencias y Enfermedades Neurodegenerativas (LINEN), Carrera de Médico Cirujano, Facultad de Estudios Superiores Iztacala, Universidad Nacional Autónoma de México, Tlalnepantla, Mexico; 2 Departamento de Neurobiología del Desarrollo y Neurofisiología, Instituto de Neurobiología, Universidad Nacional Autónoma de México, Querétaro, Mexico; 3 Posgrado en Ciencias Biológicas, Unidad de Posgrado, Edificio A, 1er Piso, Circuito de Posgrado, Ciudad Universitaria, Ciudad de México, Mexico; 4 Departamento de Fisiopatología Cardio-Renal, Instituto Nacional de Cardiología Ignacio Chávez, Ciudad de México, Mexico; 5 CECyT 6, Instituto Politécnico Nacional, Ciudad de México, Mexico; 6 División de Neurociencias, Clínica, Instituto Nacional de Rehabilitación Luis Guillermo Ibarra Ibarra, Ciudad de México, Mexico; 7 Tecnológico de Monterrey, Escuela de Medicina y Ciencias de la Salud, Ciudad de México, Mexico

**Keywords:** Alzheimer’s disease, balance, coordination, motor impairments, muscle atrophy, physical activity, physical exercise, strength

## Abstract

**Introduction:**

Alzheimer’s disease (AD) is the most common neurodegenerative disease worldwide, characterized by progressive cognitive decline and, in advanced stages, marked motor impairments. These motor deficits are associated with muscle atrophy, mitochondrial dysfunction, and amyloid-*β* (Aβ) pathology affecting both motor brain areas and peripheral tissues, ultimately contributing to disability, fall risk, and reduced quality of life. Although physical exercise has been shown to confer cognitive and functional benefits in AD, to date, no studies have directly examined the relationship between motor performance and the underlying pathological mechanisms. This study introduces a novel approach by simultaneously addressing muscle pathology and mitochondrial alterations associated with motor decline.

**Methods:**

Twelve-month-old male triple-transgenic (3xTg-AD) and non-transgenic (Non-Tg) mice were assigned to sedentary or exercise groups (*n* = 16 each group). The exercise protocol combined voluntary wheel running and forced treadmill training, 5 days/week for 4 months. Motor performance was evaluated using open-field, gait analysis, grip strength, and beam walking tests. Post-intervention, histological analyses evaluated Aβ deposition and mitochondrial morphology, biochemical assays assessed mitochondrial function, and ELISA estimated Aβ levels in the brain and muscle.

**Results:**

Physical exercise improved locomotion, balance, and strength in advanced stages of the disease, with modest benefits for memory. Histology showed reduced muscle atrophy and cortical amyloid, but not hippocampal. ELISA detected lower relative levels of Aβ only in the brain. Exercise restored reduced muscle Complex I activity, increased brain Complex IV and ATPase in both tissues, and pronounced changes in mitochondrial morphology in muscle.

**Conclusion:**

This study provides the first evidence that physical exercise improves motor function and attenuates muscle and brain pathology in advanced stages of 3xTg-AD, supporting its potential as a complementary therapeutic strategy with translational relevance to humans.

## 1 Introduction

More than 55 million people worldwide suffer from dementia, and this number is expected to triple by 2050. Of these cases, approximately 70% correspond to AD, making it the most prevalent neurodegenerative disorder globally and a major public health concern (World Health Organization, 2023). AD is characterized by two hallmark pathological features: neurofibrillary tangles composed of hyperphosphorylated tau protein and Aβ aggregates that form neuritic plaques. These pathological changes are associated with progressive cognitive decline, affecting memory, thinking, orientation, and learning abilities (Andrade-Guerrero et al., 2023c). However, AD is not solely linked to cognitive deterioration; it is also associated with a range of motor impairments that significantly impact patients’ functionality and quality of life (Koppelmans et al., 2022).

In patients with AD, a variety of motor symptoms have been identified throughout the course of the disease, including gait disturbances, reduced cardiorespiratory capacity, impaired coordination, deficits in both static and dynamic balance, increased fall risk, and muscle weakness (Ogawa et al., 2018). These alterations are evident and become exacerbated in the advanced stages of the disease (Kim et al., 2024; Poirier et al., 2021). Studies in both animal models and patients have linked several mechanisms to the onset and progression of motor symptoms. These include the spatial spread of amyloid pathology to brain regions involved in motor control, such as the motor cortex (Gupta et al., 2024). Importantly, Aβ deposition has been observed not only in the brain but also in the spinal cord and muscle fibers, suggesting a direct contribution of amyloid pathology to motor deficits (Xu H. et al., 2022). Furthermore, increased mitochondrial dysfunction at both brain and muscle levels, along with muscle atrophy, has been documented. In patients, reduced activities of mitochondrial respiratory chain complexes I and IV in the brain have been consistently reported, indicating impaired oxidative phosphorylation and energy failure (Spina et al., 2025; Li et al., 2025). Moreover, muscle atrophy is particularly significant, as it can be three to five times more severe in AD patients and is associated with reduced brain volumes (Burns et al., 2010). Collectively, these alterations contribute to a progressive decline in functionality, increased risk of disability, and higher mortality in AD patients (Andrade-Guerrero et al., 2024).

One of the most well-characterized models for studying AD is the triple-transgenic 3xTg-AD mouse, which is widely used to investigate the disease’s underlying mechanisms and identify potential therapeutic candidates. This mouse has been genetically modified to carry three mutations associated with the hereditary form of AD (PS1M146V, APPSWE, and tauP301L) (Belfiore et al., 2019; Oddo et al., 2003). Previous studies in this model have identified several motor and neuromuscular alterations, including the presence of Aβ deposition in peripheral tissues such as muscle, muscle atrophy, and denervation at the neuromuscular junction(Xu H. et al., 2022; Monteiro-Cardoso et al., 2015). Also, alterations in the activity of mitochondrial complexes I, IV, and V (ATPase) have been reported in skeletal muscle, suggesting impaired cellular energy production. This bioenergetic dysfunction may contribute to motor deterioration and the progression of AD at both the cerebral and muscular levels (Xu H. et al., 2022; Monteiro-Cardoso et al., 2015; Singulani et al., 2020). These alterations may be directly related to the motor deficits observed in this model, such as reduced locomotor activity, decreased strength, and impairments in coordination and balance, which become more evident during intermediate and late stages of the disease progression (Castillo-Mariqueo et al., 2021; Castillo-Mariqueo and Giménez-Llort, 2022a).

A complementary non-pharmacological therapeutic approach for AD is physical exercise, which encompasses a variety of planned, repetitive, and dosed physical activities that have demonstrated multiple benefits in AD (Valenzuela et al., 2020). These include reducing neuropathological markers of the disease, increasing cerebral blood flow, and promoting neuroplasticity and neurogenesis, all of which are associated with improvements in cognitive symptoms, making exercise a key complementary tool in AD treatment (López-Ortiz et al., 2021b; Andrade-Guerrero et al., 2023b). However, the mechanisms through which exercise may exert beneficial effects on motor impairments observed in the advanced stages of the disease remain incompletely understood. Therefore, this study aimed to analyze the impact of physical exercise on motor alterations, amyloid pathology, and mitochondrial function in the 3xTg-AD model. In this context, our work establishes an integrative framework focused on the muscle-brain axis, highlighting the bioenergetic and mitochondrial interplay between both tissues as a potential mechanism underlying the attenuation of motor impairments and metabolic dysfunction in AD. For the first time, a combination of behavioral, histological, biochemical, and ultrastructural analyses was used to comprehensively examine motor alterations, a key feature frequently underexplored in AD research. Our findings show that exercise not only improved locomotion, balance, and strength but also attenuated muscle atrophy, reduced cortical amyloid load, enhanced mitochondrial function in both muscle and brain, and improved mitochondrial morphology in muscle. These results highlight physical exercise as a promising complementary intervention to counteract motor disability and neuromuscular pathology in AD, with potential translational implications for patient care.

## 2 Results

### 2.1 Motor performance is lower in the 3xTg-AD model

**Figure 1:**
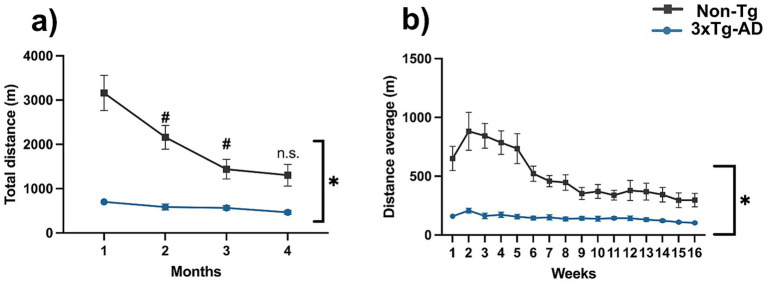
Voluntary motor activity is diminished in 3xTg-AD mice. **(a)** Distance traveled on voluntary exercise wheels (m) over 4 months. **(b)** Distance traveled per week (m) Repeated measures ANOVA, Tukey’s *post hoc*, values are means ± SEM (*n* = 16 per group), *p < 0.05 compared to the 3xTg-AD group vs. the Non-Tg group, and #*p* < 0.05 for the Non-Tg group at different months.

Regarding voluntary wheel performance, 3xTg-AD showed a significantly lower activity level compared with Non-Tg throughout the entire period (Figures 1a,b; *p* < 0.05), reflected by a reduced total distance accumulated across the 4 months (2,317 ± 185.4 m vs. 8,065 ± 993.2 m). Moreover, performance in 3xTg-AD remained unchanged during the treatment period (*p* > 0.05), whereas Non-Tg exhibited a progressive decline during the first 3 months (Figure 1a; *p* < 0.05), consistent with the gradual decrease observed in the average weekly distance (Figure 1b; *p* < 0.05).

### 2.2 Exercise increases locomotor activity and partially restores long-term memory in the 3xTg-AD model

**Figure 2:**
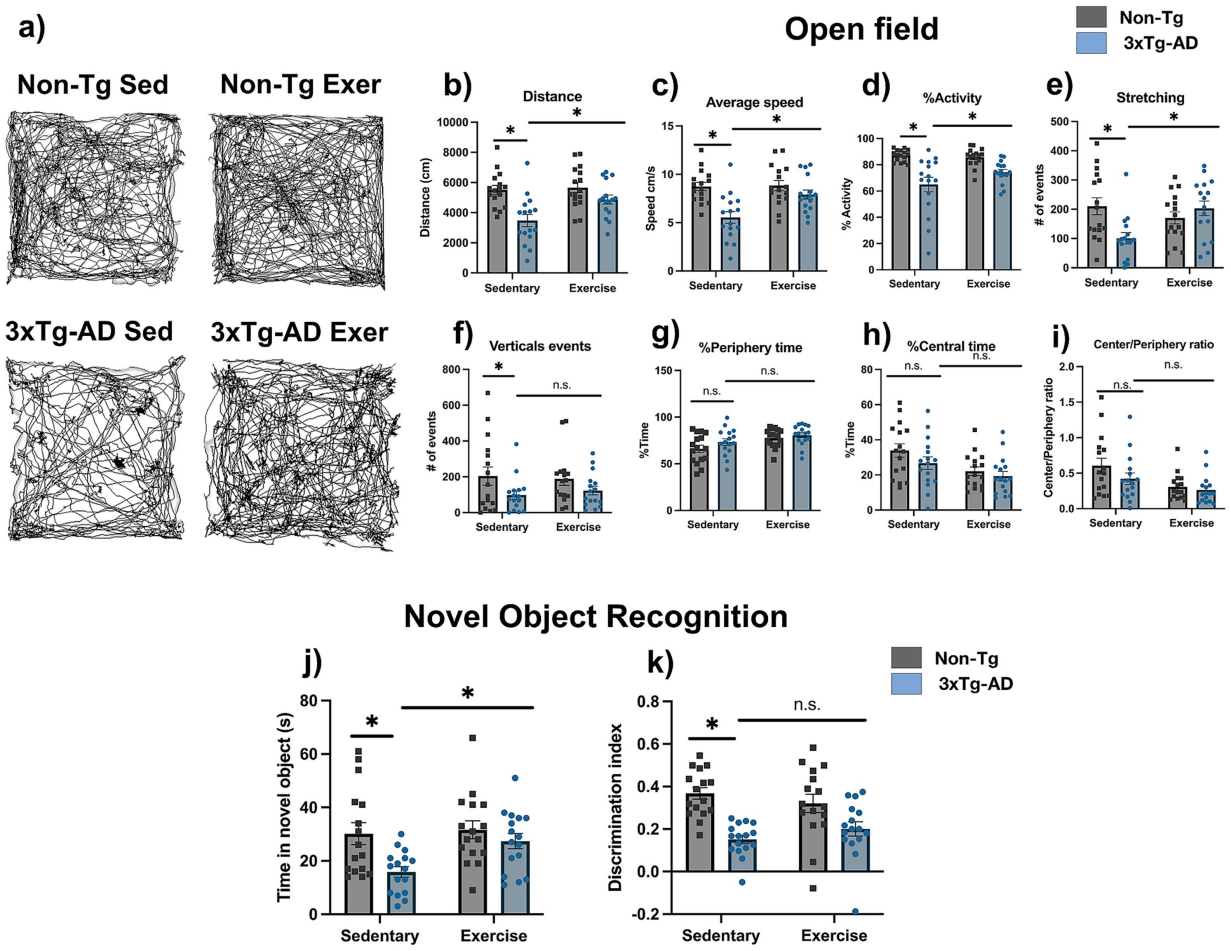
Exercise improves motor activity and partially long-term recognition memory in 3xTg-AD mice. **(a)** Representative schematics of locomotor trajectories from the different groups in the open field test. This test evaluated several parameters: **(b)** Distance traveled, **(c)** Average speed, **(d)** Percentage of activity, **(e)** Number of stretching events, **(f)** Number of vertical events, **(g)** Percentage of time spent in the periphery, **(h)** Percentage of time spent in the center, and **(i)** Center/periphery ratio. The novel object recognition evidenced: **(j)** Time exploring the novel object (s) and **(k)** Discrimination index. Statistical analyses were performed using two-way ANOVA followed by Tukey’s *post hoc* test. Data are presented as mean ± SEM (*n* = 16 per group), *p < 0.05. Exe, exercise; Sed, sedentary.

In the open field test, 3xTg-AD mice showed a significant reduction in locomotor activity compared with Non-Tg mice, with a lower distance traveled (3477.4 ± 404.3 vs. 5509.5 ± 296.3 cm), lower average speed (5.54 ± 0.60 vs. 8.75 ± 0.43 cm/s), and lower % activity (64.96 ± 5.51 vs. 87.09 ± 1.10%, Figures 2a–d, *p* < 0.05). They also showed fewer stretching and vertical events (stretching: 100.75 ± 19.26 vs. 210.38 ± 28.87; vertical events: 97.88 ± 24.30 vs. 203.56 ± 51.52; Figures 2e,f; *p* < 0.05), with no significant changes in the time spent in the periphery or center of the arena and in the center/periphery ratio (periphery: 73.27 ± 3.56 vs. 66.09 ± 3.88%; center: 26.73 ± 3.56 vs. 33.91 ± 3.88%; center/periphery ratio: 0.422 ± 0.081 vs. 0.604 ± 0.107; Figures 2g–i; *p* > 0.05). After the exercise intervention, 3xTg-AD mice showed a significant increase in distance traveled (4872.0 ± 298.0 vs. 3477.4 ± 404.3 cm), average speed (7.91 ± 0.47 vs. 5.54 ± 0.60 cm/s), % activity (74.33 ± 2.11 vs. 64.96 ± 5.51%), and number of stretching events (203.13 ± 24.91 vs. 100.75 ± 19.26, Figures 2b–e, *p* < 0.05), whereas vertical events and the time spent in the periphery and center, as well as the center/periphery ratio, remained without significant differences (vertical events: 123.13 ± 24.09 vs. 97.88 ± 24.30; periphery: 80.72 ± 2.72 vs. 73.27 ± 3.56%; center: 19.28 ± 2.72 vs. 26.73 ± 3.56%; center/periphery ratio: 0.264 ± 0.050 vs. 0.422 ± 0.081; Figures 2f–i; p > 0.05).

The novel object recognition task, 3xTg-AD mice showed impaired recognition memory compared with Non-Tg mice, with a lower time exploring the novel object (15.81 ± 2.00 vs. 30.19 ± 4.10 s) and a reduced discrimination index (0.15 ± 0.02 vs. 0.37 ± 0.03, Figures 2j,k; *p* < 0.05). After the exercise intervention, 3xTg-AD mice showed an increase in the time exploring the novel object (27.38 ± 2.84 vs. 15.81 ± 2.00 s, Figure 2j; *p* < 0.05), whereas the discrimination index remained without significant changes (0.20 ± 0.03 vs. 0.15 ± 0.02, Figure 2k, *p* > 0.05).

### 2.3 Exercise improves balance, coordination, and strength in the 3xTg-AD model

**Figure 3:**
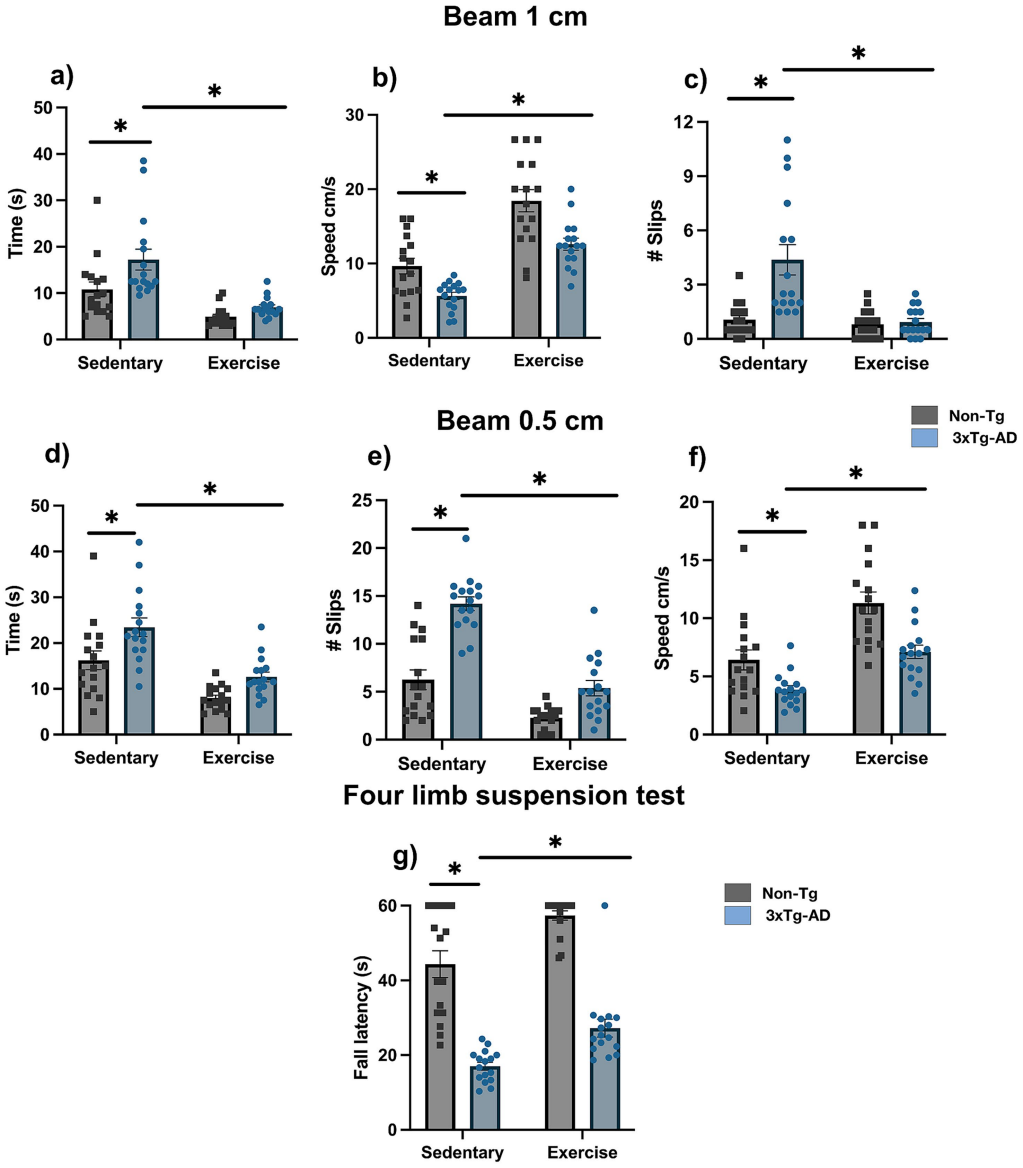
Exercise enhances balance, coordination, and strength. **(a)** Latency, **(b)** slips, and **(c)** speed on the 1 cm beam, **(d)** latency, **(e)** slips, and **(f)** speed on the 0.5 cm beam, and **(g)** latency to fall in the four limb suspension test. Statistical analyses were performed using two-way ANOVA followed by Tukey’s *post hoc* test. Data are presented as mean ± SEM (*n* = 16 per group), *p < 0.05.*

In the balance beam test, 3xTg-AD mice showed impaired coordination and balance compared with Non-Tg mice on both beams, with longer crossing times (1 cm: 17.22 ± 2.25 vs. 10.81 ± 1.60 s; 0.5 cm: 23.44 ± 2.04 vs. 16.22 ± 2.05 s), more foot slips (1 cm: 4.38 ± 0.84 vs. 1.06 ± 0.23; 0.5 cm: 14.19 ± 0.72 vs. 6.25 ± 1.02), and lower speeds (1 cm: 5.65 ± 0.48 vs. 9.66 ± 1.05 cm/s; 0.5 cm: 3.85 ± 0.35 vs. 6.42 ± 0.86 cm/s; Figures 3a–f; *p* < 0.05). In addition, 3xTg-AD mice exhibited reduced muscular strength, reflected by a shorter latency to fall in the four-limb suspension test (17.04 ± 1.05 vs. 44.33 ± 3.57 s; Figure 3g; *p* < 0.05). After the exercise intervention, 3xTg-AD mice showed improved performance on both beams, with reduced time (1 cm: 6.94 ± 0.53 vs. 17.22 ± 2.25 s; 0.5 cm: 12.59 ± 1.07 vs. 23.44 ± 2.04 s), fewer slips (1 cm: 0.94 ± 0.20 vs. 4.38 ± 0.84; 0.5 cm: 5.38 ± 0.80 vs. 14.19 ± 0.72), and increased speed (1 cm: 12.61 ± 0.81 vs. 5.65 ± 0.48 cm/s; 0.5 cm: 7.11 ± 0.58 vs. 3.85 ± 0.35 cm/s; Figures 3a–f; *p* < 0.05), along with increased strength as indicated by a longer latency to fall (27.23 ± 2.40 vs. 17.04 ± 1.05 s; Figure 3g; *p* < 0.05).

### 2.4 Exercise mitigates sarcopenic and adiposity alterations in the 3xTg-AD model

**Figure 4:**
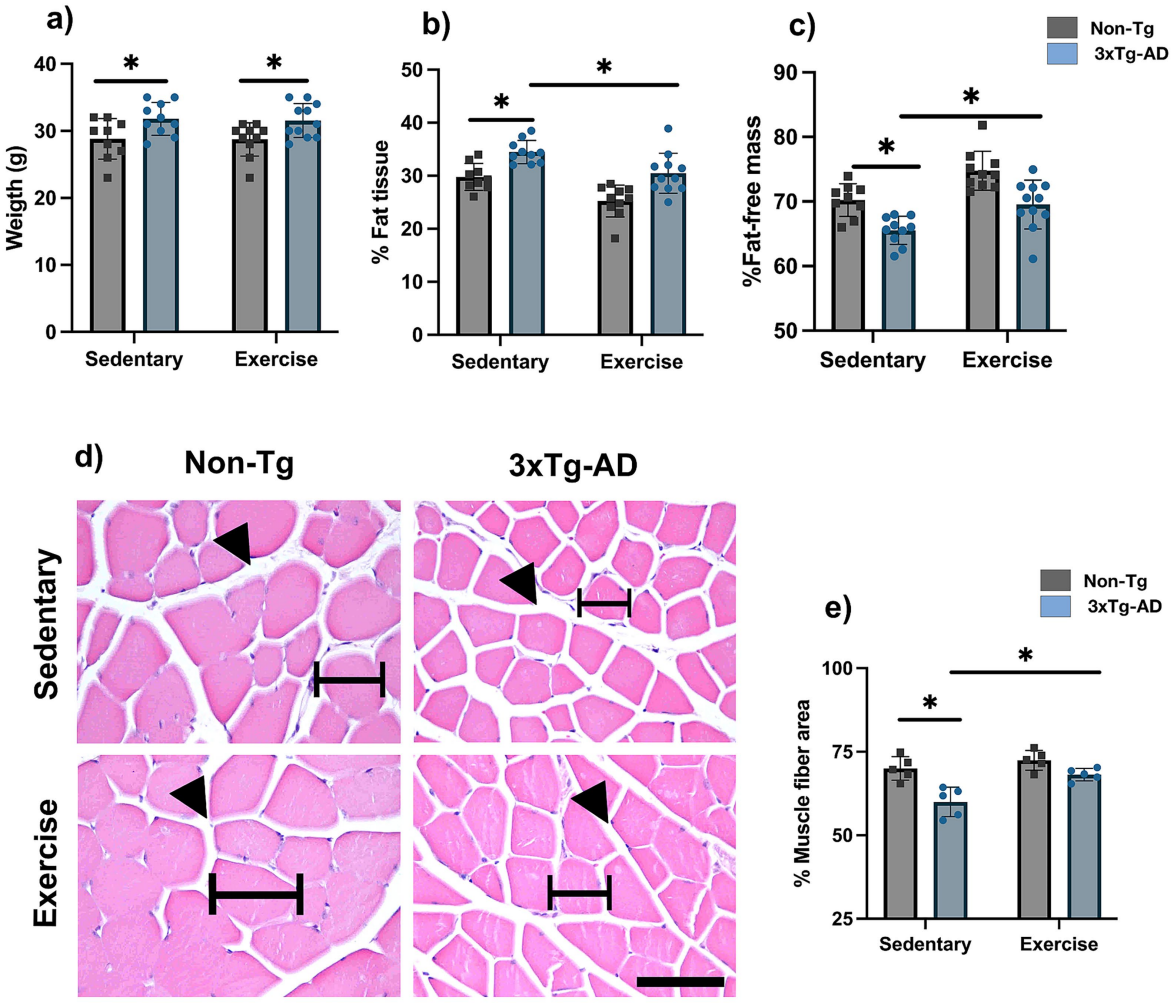
Exercise attenuates muscle loss and fat accumulation in 3xTg-AD mice. **(a)** Body weight, **(b)** Percentage of adipose tissue, **(c)** Fat-free tissue, **(d)** Representative images of gastrocnemius muscle tissue under different conditions stained with H&E, and **(e)** Muscle area percentage measured by bioimpedance analysis. For body weight and bioimpedance analysis, *n* = 10–11 per group, and for histology, *n* = 5 per group. Statistical analyses were performed using two-way ANOVA followed by Tukey’s post hoc test. Data are presented as mean ± SEM, *p* < 0.05. Scale bar = 50 μm for 40x magnification (common to all microphotographs). Black arrowheads indicate representative muscle fibers, and black bars highlight the endomysial space (intramuscular connective tissue), illustrating differences in fiber size and connective tissue between the different conditions.

In anthropometric parameters, 3xTg-AD mice showed a higher body weight compared with Non-Tg mice (31.80 ± 0.77 vs. 28.78 ± 1.01 g), together with a higher fat tissue percentage (34.46 ± 0.69 vs. 29.78 ± 0.84%) and a lower fat-free mass percentage (65.54 ± 0.69 vs. 70.22 ± 0.84%; Figures 4a–c; *p* < 0.05). After the exercise intervention, body weight remained unchanged (31.55 ± 0.77 vs. 31.80 ± 0.77 g; Figure 4a; *p* > 0.05), whereas fat tissue percentage decreased (30.47 ± 1.14 vs. 34.46 ± 0.69%) and fat-free mass percentage increased (69.54 ± 1.14 vs. 65.54 ± 0.69%; Figures 4b,c; *p* < 0.05). Histological analysis showed a smaller muscle fiber area in 3xTg-AD mice compared with Non-Tg mice (60.00 ± 1.96 vs. 69.99 ± 1.58%), and exercise increased muscle fiber area (68.15 ± 0.83 vs. 60.00 ± 1.96%; Figures 4d,e; *p* < 0.05).

### 2.5 Physical exercise selectively exerts region-specific effects on amyloid pathology in the 3xTg-AD model

**Figure 5:**
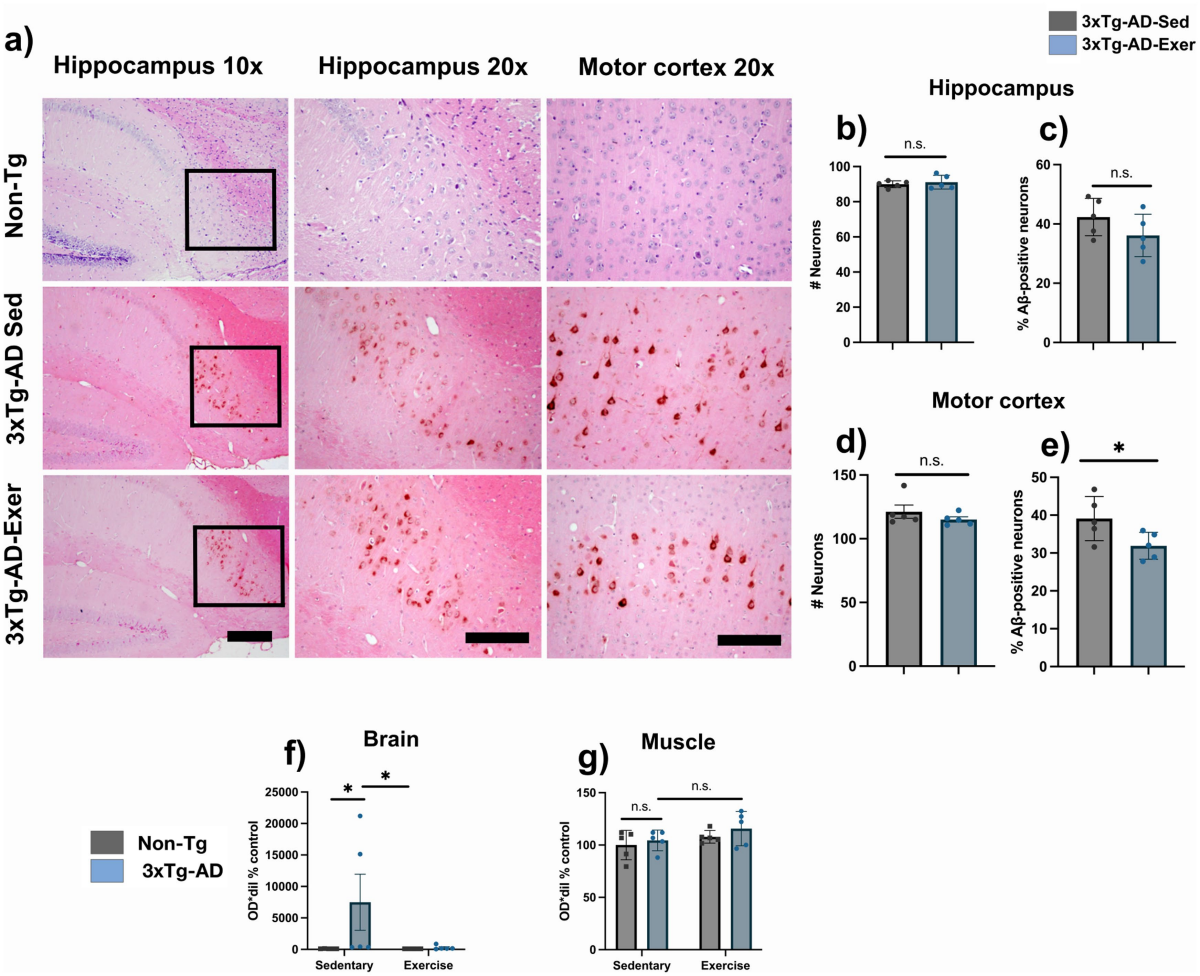
Region-selective reduction of amyloid pathology induced by exercise in 3xTg-AD mice. **(a)** Representative microphotographs of the hippocampus and motor cortex under different conditions. **(b)** Number of neurons in the hippocampus. **(c)** Percentage of Aβ-positive cells in the hippocampus. **(d)** Number of neurons in the motor cortex. **(e)** Percentage of Aβ-positive cells in the motor cortex. **(f,g)** Relative Aβ1-42 levels in the muscle and one hemisphere of the brain. Statistical analyses were performed using Student’s *t*-test, ANOVA, and the Kruskal-Wallis test. Data are presented as mean ± SEM, *n* = 5 per group, *p* < 0.05. Scale bar = 100 μm for x10 magnification and 50 μm for x20 magnification (applies to all microphotographs).

The exercise intervention did not cause a significant decrease in the total number of neurons, nor of Aβ-positive neurons, in the hippocampus (Figures 5a–c; 89.93 ± 0.85 vs. 91.13 ± 1.77 for total neurons; 42.33 ± 2.83 vs. 36.11 ± 3.19 for Aβ-positive neurons; *p* > 0.05). In the motor cortex, neuronal density also remained unchanged (Figures 5a,d; 121.20 ± 5.20 vs. 115.10 ± 2.06; *p* > 0.05), whereas the percentage of Aβ-positive neurons was significantly reduced after exercise (Figures 5a,e; 39.11 ± 2.61 vs. 31.91 ± 1.59; *p* < 0.05). ELISA analyses showed that brain relative Aβ-42 levels were elevated only in sedentary 3xTg-AD compared with the other groups (Figure 5f; 7486.30 ± 4462.93 vs. 100.00 ± 7.59; *p* < 0.05), and a decrease was observed after exercise without reaching statistical significance (Figure 5f; 7486.30 ± 4462.93 vs. 272.60 ± 150.55; *p* > 0.05). In muscle, no significant changes in Aβ1-42 were detected between groups or after exercise (Figure 5g; 100.00 ± 6.28 and 107.86 ± 2.72;104.44 ± 4.41 and 115.73 ± 7.32; *p* > 0.05).

### 2.6 Exercise-induced restoration of mitochondrial complex activities in the brain and muscle of 3xTg-AD mice

**Figure 6:**
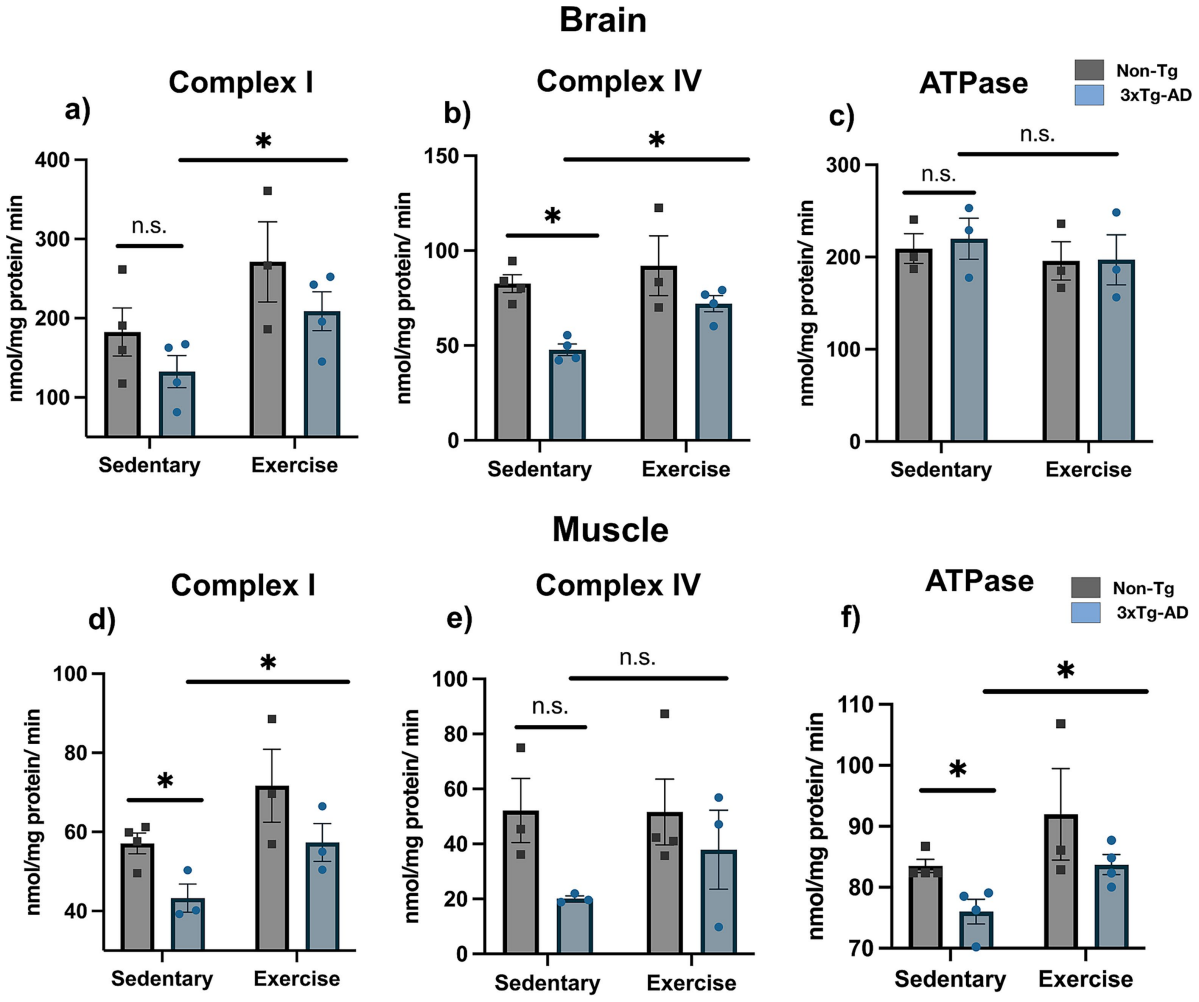
Exercise improves mitochondrial complex activity in the brains and skeletal muscles of 3xTg-AD mice. Complex I activity in the brain **(a)** and muscle tissue **(d)**. Complex IV activity in the brain **(b)** and muscle tissue **(e)**. ATPase activity in brain **(c)** and muscle tissue **(f)**. Statistical analyses were performed using two-way ANOVA followed by Tukey’s *post hoc* test. Data are presented as mean ± SEM, *n* = 3–4 per group, *p < 0.05.

In brain 3xTg-AD mice showed no changes in complex I activity compared with Non-Tg mice (182.477 ± 30.362 vs. 132.560 ± 20.215 nmol/min/mg protein; Figure 6a; *p* > 0.05). In contrast, 3xTg-AD mice showed lower complex IV activity than Non-Tg mice (82.649 ± 4.725 vs. 47.781 ± 3.077 nmol/min/mg protein; Figure 6b; *p* < 0.05), whereas ATPase activity showed no significant differences between groups (209.200 ± 16.100 vs. 219.867 ± 22.279 nmol/min/mg protein; Figure 6c; *p* > 0.05). After the exercise intervention, in 3xTg-AD mice complex I activity increased (132.560 ± 20.215 vs. 208.897 ± 24.543 nmol/min/mg protein; Figure 6a; *p* < 0.05), whereas complex IV activity increased (47.781 ± 3.077 vs. 72.085 ± 4.233 nmol/min/mg protein; Figure 6b; *p* < 0.05); ATPase activity showed no significant changes (219.867 ± 22.279 vs. 197.033 ± 27.113 nmol/min/mg protein; Figure 6c; *p* > 0.05).

In skeletal muscle 3xTg-AD mice showed reduced complex I activity compared with Non-Tg mice (57.087 ± 2.609 vs. 43.258 ± 3.555 nmol/min/mg protein; Figure 6d; *p* < 0.05), whereas complex IV activity showed no significant differences between groups (52.187 ± 11.688 vs. 20.127 ± 0.947 nmol/min/mg protein; Figure 6e; *p* > 0.05). In addition, 3xTg-AD mice showed lower ATPase activity than Non-Tg mice (83.450 ± 1.080 vs. 76.016 ± 2.026 nmol/min/mg protein; Figure 6f; *p* < 0.05). After the exercise intervention, in 3xTg-AD mice complex I activity increased (43.258 ± 3.555 vs. 57.322 ± 4.765 nmol/min/mg protein; Figure 6d; *p* < 0.05) and ATPase activity also increased (76.016 ± 2.026 vs. 83.718 ± 1.649 nmol/min/mg protein; Figure 6f; *p* < 0.05), with no significant changes in complex IV activity (20.127 ± 0.947 vs. 37.923 ± 14.361 nmol/min/mg protein; Figure 6e; *p* > 0.05).

### 2.7 Exercise induces improvements in mitochondrial muscle morphology

**Figure 7:**
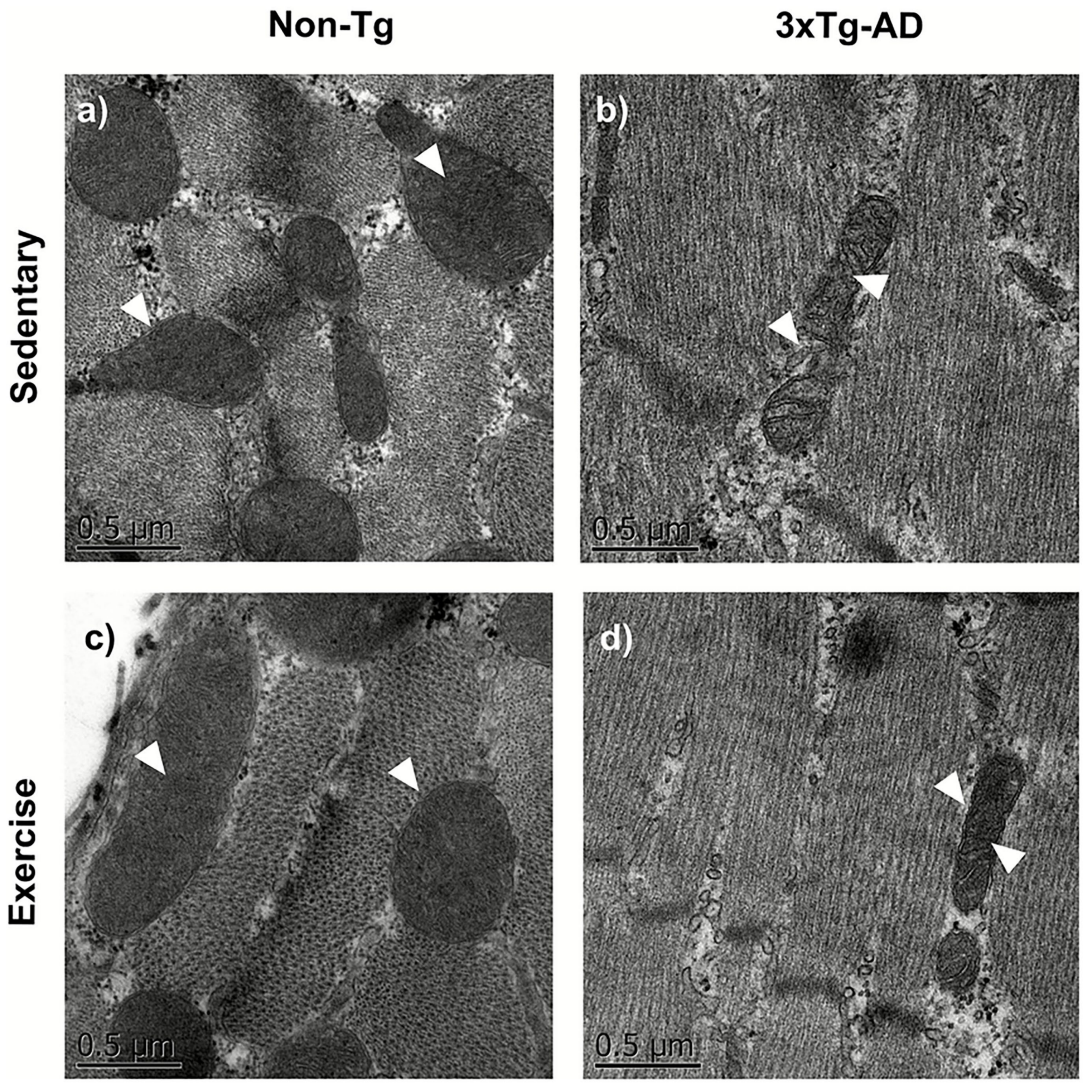
Physical exercise restores muscle mitochondrial structure in the 3xTg-AD model. Transmission electron microscopy images are shown, where structural differences (white arrowheads) can be observed across different conditions: **(a)** Non-Tg sedentary, **(b)** Non-Tg exercise, **(c)** 3xTg-AD sedentary, **(d)** 3xTg-AD exercise.

The analysis of mitochondrial morphology was qualitative and based on the inspection of representative transmission electron microscopy images. In sedentary Non-Tg mice, muscle mitochondria exhibited a physiological morphology characterized by elongated and preserved organization. After the exercise intervention, the mitochondria in this group showed further elongation (Figures 7a,b).

In sedentary 3xTg-AD mice, mitochondria exhibited marked ultrastructural alterations, appearing more rounded and less organized. However, after exercise, mitochondria in 3xTg-AD mice showed an overall improvement in morphological appearance and organization (Figures 7c,d). Based on qualitative observation only, mitochondrial profiles appeared less frequent per field in sedentary 3xTg-AD mice than in Non-Tg mice, whereas exercise was associated with an apparent increase in mitochondrial profiles.

### 2.8 Muscle strength correlates with amyloid pathology and body composition changes

**Figure 8:**
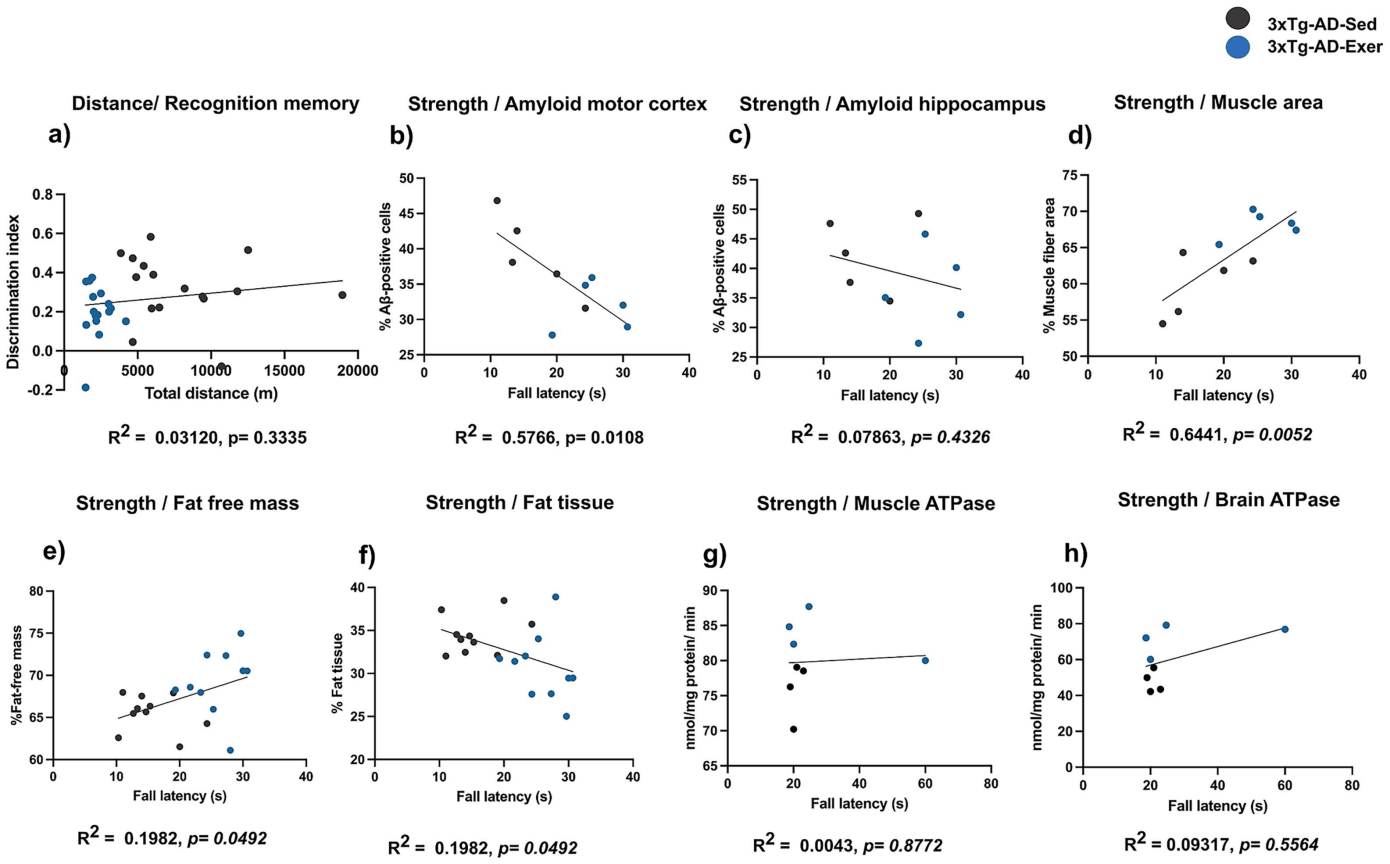
Correlation between muscle strength and amyloid pathology, body composition, and ATPase activity in 3xTg-AD mice. The graphs show the correlation between total distance run in the voluntary wheel and performance in the novel object recognition test **(a)**, and between grip strength and amyloid in the motor cortex **(b)** and hippocampus **(c)**, muscle fiber area **(d)**, fat-free mass **(e)**, fat tissue **(f)**, muscle ATPase activity **(g)**, and brain ATPase activity **(h)**. Pearson’s correlation coefficient and linear regression are indicated in each graph. *p* < 0.05 was considered a statistically significant difference.

No significant correlation was observed between the total distance run in the voluntary wheel over the entire 4-month intervention period and performance in the novel object recognition test (*R*^2^ = 0.0312, *p* = 0.3335; Figure 8a). Muscle strength correlated with amyloid pathology, body composition, and ATPase activity in 3xTg-AD mice (Figure 8). A significant negative correlation was observed between grip strength and amyloid burden in the motor cortex (*R*^2^ = 0.5766, *p* = 0.0108; Figure 8a), whereas no association was found in the hippocampus (*R*^2^ = 0.0786, *p* = 0.4326; Figure 8b). In contrast, muscle strength showed a strong positive correlation with muscle fiber cross-sectional area (*R*^2^ = 0.6441, *p* = 0.0052; Figure 8c) and a positive association with fat-free mass (*R*^2^ = 0.1982, *p* = 0.0492; Figure 8d). Conversely, a negative correlation was detected between muscle strength and fat tissue percentage (*R*^2^ = 0.1982, p = 0.0492; Figure 8e). No significant relationships were found between muscle strength and muscle ATPase activity (*R*^2^ = 0.0043, *p* = 0.8772; Figure 8f) or brain ATPase activity (*R*^2^ = 0.2419, *p* = 0.2157; Figure 8g). No significant correlations were found between locomotor or balance activity and amyloid pathology or body composition. A moderate, no significant association was detected with mitochondrial ATPase activity in muscle and brain (see Supplementary material).

## 3 Materials and methods

### 3.1 Animals and experimental groups

For this study, the triple-transgenic mouse model of Alzheimer’s disease (3xTg-AD) was used, which carries the *APPSwe* and *tauP301L* transgenes in a *PS1M146V* knock-in background (Oddo et al., 2003). A total of 64 twelve-month-old male mice were randomly and evenly assigned to four experimental groups: 3xTg-AD sedentary (*n* = 16), 3xTg-AD exercise (*n* = 16), Non-Tg sedentary (*n* = 16), and Non-Tg exercise (*n* = 16). Only male 3xTg-AD mice were used to minimize endocrine influences on mitochondrial bioenergetics and exercise responsiveness. Males of this strain exhibit a more pronounced motor and frailty phenotype, with progressive loss of strength and coordination (Dennison et al., 2021; Castillo-Mariqueo and Giménez-Llort, 2022b; Kane et al., 2018), consistent with prior reports describing gait impairment and sarcopenia (Castillo-Mariqueo et al., 2021). All animals were genotyped before starting the study. Mice were housed in groups of 4–5 per cage, with each cage containing animals of the same genotype. They had *ad libitum* access to food and water under controlled conditions (12:12 h light–dark cycle, temperature 22 ± 2 °C, relative humidity of 60 ± 5%). Animal handling was conducted under veterinary supervision. The study was performed in accordance with the NIH Guide for the Care and Use of Laboratory Animals. It was approved by the Bioethics Committee of the Institute of Neurobiology, National Autonomous University of Mexico (protocol number 117).

### 3.2 Behavioral tasks

#### 3.2.1 Experimental paradigm

**Figure 9:**
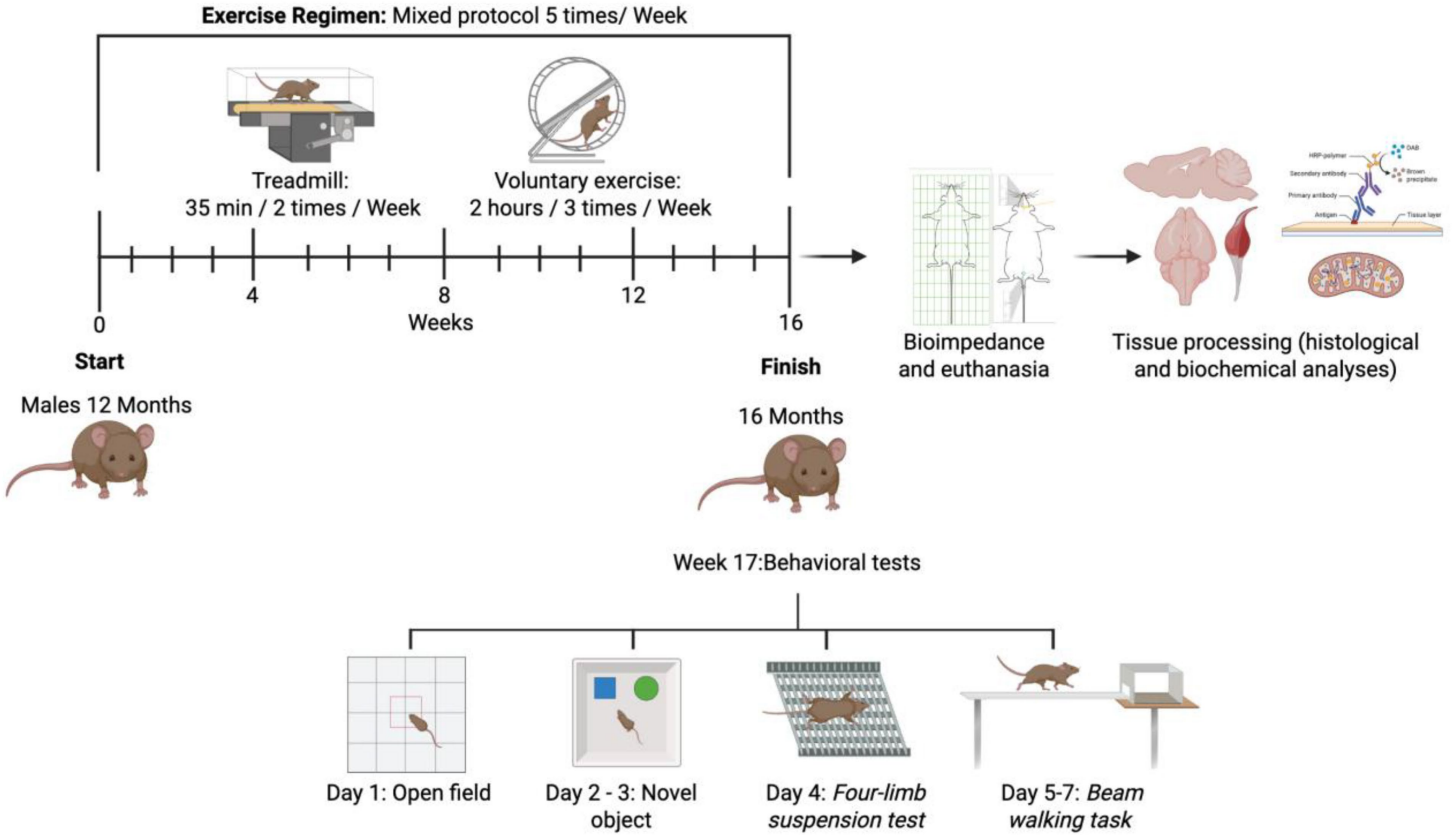
Schematic illustrating the experimental timeline. Animals entered the study at 12 months of age, underwent 16 weeks of physical exercise, and were subsequently subjected to behavioral tests and bioimpedance analysis. After euthanasia, muscle and brain tissues were dissected for subsequent experimental procedures and data analysis.

The experimental paradigm (Figure 9) shows that the exercise protocol was conducted from 12 to 16 months of age, a period encompassing intermediate-to-advanced phases of pathological progression in the 3xTg-AD model. During this interval, a progressive increase in neuropathological signs, as well as synaptic, mitochondrial, and motor alterations characteristic of advanced stages of the disease, has been described (Belfiore et al., 2019; Castillo-Mariqueo et al., 2021). At 12 months old, male mice were subjected to physical exercise using a mixed program for 4 months, followed by motor tests, bioimpedance measurements, euthanasia, and sample processing. Behavioral testing and histological analyses were conducted under randomized and blinded conditions. Animals were randomly assigned to experimental groups, and the investigators performing behavioral scoring and tissue quantification were blinded to group identity throughout data collection and analysis.

#### 3.2.2 Exercise regimen

The groups assigned to the exercise condition underwent a 16-week program combining voluntary and forced exercise. This mixed paradigm was designed to integrate the complementary benefits of both modalities: voluntary running allows spontaneous physical activity, whereas forced treadmill exercise provides controlled intensity and duration (Andrade-Guerrero et al., 2023b). Voluntary exercise was performed three times per week on an exercise wheel (Panlab LE905, Barcelona, Spain). Mice were allowed free access to the wheel for 2 h per session. Session duration was restricted to 2 h to maintain higher training density and minimize inter-individual variability in voluntary running exposure. Wheel rotations were detected by a sensor mounted on top of the cage and transmitted to a PC running dedicated software, which recorded the distance traveled per animal per day in kilometers. From these recordings, voluntary wheel activity was quantified as the total distance traveled per month (corresponding to the sum of weekly distances) and as the average distance traveled per week, both of which were used for data analysis and graphical representation. Forced exercise was conducted twice per week on a custom-adapted treadmill (TM008-BK, China) for 35 min per session. A gradual speed adaptation protocol was implemented during the first 4 weeks: 7 m/min (week 1), 9 m/min (week 2), 10 m/min (week 3), and 11 m/min (week 4). This progressive adaptation was designed to improve treadmill tolerance, minimize stress, and reduce the risk of excessive fatigue. From week 5 to week 16, animals underwent training at a constant speed of 13 m/min. These treadmill speeds correspond to an estimated range of approximately 55–80% VO₂max, based on previously established correlations between treadmill speed and oxygen consumption in mice (Fernando et al., 1993; Schefer and Talan, 1996), rather than on direct individual VO₂max measurements. This range is considered to represent moderate-to-vigorous exercise intensity, consistent with current physical activity recommendations (WHO Guidelines on Physical Activity and Sedentary Behaviour, 2020).

#### 3.2.3 Locomotor activity and long-term memory

Locomotor activity and cognition were evaluated 72 h after the last training session using the Open Field Test (OFT) and the Novel Object Recognition (NOR) task (Leger et al., 2013; Seibenhener and Wooten, 2015). All animals underwent both tests. To prevent olfactory cues, the arena was cleaned with 70% ethanol between trials, and all experiments were conducted at a fixed time under controlled lighting and temperature. On day 1, each animal was placed in an acrylic box (33 × 33 × 33 cm) and positioned at the center of the open field, and allowed to explore freely for 10 min. The following variables were recorded: total distance traveled, average speed, maximum speed, percentage of active time, number of vertical events and stretches, and percentage of time spent in the center and periphery. The NOR task was conducted on days 2 and 3. On day 2, two identical objects were placed in the corners of the arena, and animals were allowed to explore them freely for 10 min. After a 24-h interval, one of the objects was replaced with a novel object, and animals were again allowed to explore freely for 5 min. The time spent exploring each object was recorded, and a discrimination index (DI) was calculated as (T_novel − T_familiar)/(T_novel + T_familiar), where T_novel and T_familiar denote the time spent exploring the novel and familiar object, respectively. All sessions were video-recorded and analyzed using the Smart Video Tracking 2.5 software (Panlab, Barcelona, Spain).

#### 3.2.4 Four-limb suspension test

To evaluate strength and endurance in mice, the four-limb suspension test was used (Osmon et al., 2018). Mice were placed in the center of a wire mesh grid (2 mm diameter) suspended 40 cm above the ground, with a cushioned surface below to prevent injury. The animals were allowed to grasp the mesh with all four limbs. Once a secure grip was established, the mesh was gently inverted, leaving the mice suspended upside down. The fall latency was recorded using a stopwatch, with a maximum of 60 s per trial. Three trials were performed for each mouse with a minimum rest period of 2 min between trials to prevent fatigue, and the average latency across the three trials was calculated.

#### 3.2.5 Beam-walking task

To assess balance and motor coordination, the beam-walking task was performed (Luong et al., 2011; Ito et al., 2022). Two beams, each 1 meter in length and with widths of 0.5 cm or 1 cm, were elevated 50 cm above the ground. Consistent with most established beam-walking protocols, we used two beam diameters to provide graded task difficulty, minimize floor and ceiling effects, and increase sensitivity to subtle balance and coordination deficits. Animals were trained for two consecutive days before the final evaluation, completing two trials per day on each beam. On day 3 (test phase), each mouse was placed at one end of the beam and underwent two test trials per beam. The following variables were recorded: average crossing time, speed, and number of foot slips.

### 3.3 Bioimpedance analysis

After the animals were anesthetized, but before euthanasia, body composition was assessed by bioimpedance analysis using the ImpediVET Vet BIS1 device (ImpediMed, Brisbane, Australia), a single-channel, four-electrode bioimpedance spectroscopy (BIS) system (Chapman et al., 2010). This technique was used to estimate whole-body fat mass (FM) and fat-free mass (FFM) percentages. The device scans 256 frequencies between 4 kHz and 1,000 kHz.

Electrodes were placed in a standardized tetrapolar configuration: one electrode along the midline of the back, following an imaginary line connecting the ears with the nose; a second electrode 1 cm anterior to the first; a third electrode along the midline of the back from the intersection of the thigh muscles with the body toward the midline; and a fourth electrode positioned 2 cm posterior to the third electrode. This configuration ensured accurate impedance measurements for calculating FM and FFM, which were used for subsequent body composition analyses.

### 3.4 Tissue fixation and sectioning for histological and molecular analyses

Animals were anesthetized with a mixture of ketamine (100 mg/kg) and xylazine (10 mg/kg) administered intraperitoneally. They were then euthanized via transcardial perfusion with a 4% paraformaldehyde (PFA) solution in phosphate-buffered saline (PBS) containing 1,000 IU of heparin and 0.1% procaine. The brains and muscles were extracted and fixed for 24 h in a 4% buffered PFA solution at room temperature. Subsequently, the brains were embedded in paraffin, and serial coronal sections (5 μm thickness) were obtained using a sliding microtome (Leica RM 2135, Wetzlar, Germany) to access the hippocampal region, with sagittal cuts performed at lateral levels 1.9 to 2.9 mm according to the Paxinos atlas. For fresh tissue analysis, a rapid extraction protocol was used: brains and muscles were removed immediately after euthanasia, frozen at −70 °C, and processed for molecular studies.

### 3.5 Immunohistochemistry assays

The tissues were deparaffinized at 60 °C, rehydrated, and incubated with 1% hydrogen peroxide. Antigen retrieval was performed in a citrate buffer for 10 min using a microwave, followed by incubation in a blocking solution containing 0.2% bovine serum albumin (Sigma-Aldrich, St. Louis, MO, USA) for 1 h. Subsequently, the primary antibody anti-mouse Aβ (BAM-10, 1:500, Sigma-Aldrich, St. Louis, MO, USA) was incubated overnight at 4 °C in a humid chamber. The following day, tissues were incubated with the corresponding biotinylated secondary antibody (1:500, Invitrogen Molecular Probes, Eugene, OR, USA) for 2 h at room temperature and then revealed using the ABC/diaminobenzidine kit. Finally, these tissues were counterstained with hematoxylin–eosin (H&E) and subsequently mounted on slides using Entellan resin (Merck, KGaA, Darmstadt, Germany). In the case of muscle tissue, only H&E staining was used for the analysis of muscle fibers. Muscle sections (5 μm) were deparaffinized in xylene, rehydrated through a graded series of alcohols, stained with Mayer’s hematoxylin for 2 min, washed with running water, counterstained with eosin for 15 s, dehydrated in ethanol, and mounted with Entellan resin. Image analysis and quantification were performed using ImageJ software (NIH, Bethesda, MD, USA). For each animal, four random histological levels were analyzed in both brain and muscle tissue. In the brain, sections corresponding to lateral coordinates between 1.4 and 3.4 mm were evaluated according to the Paxinos and Watson atlas. The quantification included the neuron count per field and the percentage of Aβ-positive neurons within the subiculum and motor cortex. For muscle tissue, four representative levels were analyzed from random sections of the gastrocnemius belly, and the percentage of total muscle area was determined using a threshold function to delineate tissue boundaries and calculate the proportion of stained area.

### 3.6 Ultrastructural analysis of mitochondria in skeletal muscle

Fragments of the gastrocnemius muscle were fixed in 2.5% glutaraldehyde (Electron Microscopy Sciences, Hatfield, PA, USA) in 0.1 M phosphate buffer (pH 7.4) for 24 h at 4 °C, and subsequently post-fixed in 1% osmium tetroxide (Electron Microscopy Sciences, Hatfield, PA, USA) for 1 h at room temperature. Samples were dehydrated through a graded ethanol series and embedded in epoxy resin (EMbed 812, Electron Microscopy Sciences, Hatfield, PA, USA).

Ultrathin sections (~70 nm) were obtained using an RMC MT-X ultramicrotome (Boeckeler Instruments, Tucson, AZ, USA), mounted on copper grids, and contrasted with 2% uranyl acetate and 0.2% lead citrate. Observations were performed with a JEOL JEM-1010 transmission electron microscope (JEOL Ltd., Tokyo, Japan) operating at 60–80 kV. Images were acquired with a Gatan Bioscan 1 K × 1 K digital camera using Digital Micrograph 3.1 software (Gatan, Pleasanton, CA, USA). Electron microscopy was conducted as a qualitative ultrastructural assessment, and representative images were obtained from one animal per experimental group.

### 3.7 ELISA assays

Brain and muscle tissues were processed using standard protocols. Brain samples were homogenized in 8 volumes of cold 5 M guanidine-HCl in 50 mM Tris buffer with a Dounce homogenizer, mixed for 4 h at room temperature, diluted 1:10 with cold PBS containing protease inhibitor cocktail (Thermo Scientific™, cat# 78429), and centrifuged at 16,000 × g for 20 min at 4 °C. Muscle samples were homogenized in 8 volumes of Cell Extraction Buffer (Thermo Scientific™, cat# FNN0011) with protease inhibitor cocktail, vortexed, incubated on ice for 30 min, and centrifuged under the same conditions. Supernatants were collected, aliquoted, and stored at −80 °C.

Relative Aβ1-42 levels were measured by sandwich ELISA (Abcam, cat# ab253583). High-binding plates were coated overnight at 4 °C with capture antibody, blocked with 1% BSA in PBS for 1 h, and loaded with diluted samples in duplicate (100 μL/well) for 60 min at room temperature. After washing, plates were incubated with detection antibody (60 min) and HRP-conjugated anti-rabbit secondary antibody (Vector Laboratories, cat# PI-1000, 30 min). Color development was performed using TMB substrate (30 min, dark), stopped with 1 M H₂SO₄, and absorbance was read at 450 nm (BioTek Epoch). Aβ1-42 levels were expressed as OD values relative to non-transgenic sedentary controls, corrected for dilution (OD*dil% control).

### 3.8 Mitochondrial analysis

Mitochondrial respiratory complex activities were assessed spectrophotometrically using a Cytation 7 reader (Biotek Instruments, Winooski, VT, USA). Mitochondrial fractions were isolated from whole-brain and gastrocnemius muscle homogenates. Tissues were homogenized on ice using a Potter-type homogenizer in isolation buffer and centrifuged at 2,060 g to remove nuclei and cellular debris. The supernatant was collected and subjected to two successive centrifugations at 10,000 g in the same isolation buffer, retaining the mitochondrial pellet at each step. The final pellet was resuspended in 200 μL of isolation buffer, and this preparation was used as the source of mitochondrial protein for the enzymatic assays. Complex I activity was measured in microplates containing 0.5 μg of mitochondrial protein in Mir05 buffer supplemented with DCPIP, NADH, antimycin A, and sodium azide, with the reaction initiated by decylubiquinone (3.2 mM). Absorbance at 600 nm was monitored for 3 min at 37 °C, and rotenone (13 μM) was used as a specific inhibitor. Complex IV activity was evaluated with 0.25 μg of mitochondrial protein in Mir05 buffer containing reduced cytochrome c (17 μM) and Tween-20 (220 μM), and the oxidation of cytochrome c was monitored at 550 nm. Sodium azide (5 mM) was used to determine specific activity. ATP synthase activity was indirectly quantified by coupling ATP hydrolysis to NADPH synthesis at 340 nm, using hexokinase and glucose-6-phosphate dehydrogenase. After baseline readings, ADP (1.2 mM) was added to initiate ATP synthesis, and CCCP (0.05 μM) was added to inhibit it, with kinetics recorded at 37 °C. Results were expressed as nmol/min/mg protein.

### 3.9 Statistical analysis

The data were normalized and expressed as mean ± standard error of the mean (S. E. M.). For statistical analysis, Student’s t-test, two-way analysis of variance (ANOVA), repeated-measures ANOVA, and the Kruskal-Wallis test were applied, followed by group comparisons using Tukey’s *post hoc* test. Data processing and graph generation were performed using PRISMA 10 software, with a significance level of *p* ≤ 0.05. In addition, correlation analyses were performed for the sedentary and exercise groups of 3xTg-AD mice, using Pearson’s correlation coefficient and linear regression to assess relationships between variables. Statistical significance was accepted at *p* < 0.05.

## 4 Discussion

This study demonstrates that at 16 months of age (advanced stages of the pathology), 3xTg-AD mice exhibit multiple motor impairments, including reduced physical performance and locomotor activity, balance, strength, and coordination. These alterations are associated with mitochondrial dysfunction at both the brain and muscle levels, as well as with the presence of amyloid pathology in the motor cortex and skeletal muscle. After a mixed voluntary and forced exercise program, we observed improvements in several motor parameters, partial restoration of mitochondrial and cognitive function, and a region-specific reduction in amyloid pathology in motor areas.

Building on these findings, our open-field data revealed marked reductions in locomotor activity and spontaneous exploration in sedentary 3xTg-AD mice compared with Non-Tg controls. This pattern, reflected by shorter distances traveled during voluntary wheel running and lower open-field measures (distance and speed), indicates a progressive deterioration in motor performance and reduced motivation to explore the environment. Notably, the decline in wheel-running distance observed after week 5 occurred in Non-Tg control mice under physiological conditions, likely reflecting habituation to the wheel and natural adaptation to voluntary running. In contrast, 3xTg-AD mice exhibited consistently reduced motor performance from the earliest time points, which remained relatively stable throughout the experimental period rather than deteriorating further. These results are consistent with previous studies reporting diminished locomotor activity and increased apathy in this model (Andrade-Guerrero et al., 2023a; Szabó et al., 2023) and highlight the combined impact of central and peripheral impairments on motor behavior (Pentkowski et al., 2021; Castillo-Mariqueo et al., 2021).

After the mixed voluntary/forced exercise intervention, 3xTg-AD mice displayed significant improvements in locomotor activity, coordination, balance, and strength, supporting evidence that exercise can induce beneficial adaptations even at advanced stages (Lee et al., 2023; Nakanishi et al., 2021; Enette et al., 2020). Notably, these improvements occurred without detectable cognitive changes, contrasting with most published studies reporting cognitive benefits in earlier disease stages (Kim et al., 2019; Xu B. et al., 2022; Andrade-Guerrero et al., 2023b). The lack of robust cognitive improvement in this study is likely due to the advanced disease stage, in which severe damage to hippocampal and cortical networks limits the cognitive response to exercise. To date, very few studies have examined the effects of exercise at such late stages. In this context, the beneficial effects of exercise on the locomotor system may occur largely independently of cognitive performance, through central and peripheral mechanisms related to neuromuscular control.

Regionally, we observed a significant reduction in A*β*-positive cells and amyloid burden in the motor cortex, but not in the hippocampus, after exercise. Most studies report hippocampal decreases in earlier disease stages (Valenzuela et al., 2020; López-Ortiz et al., 2021a), but the high structural damage of the hippocampus at 16 months likely limits exercise effects (Bartsch and Wulff, 2015). In contrast, the motor cortex, directly engaged during physical training, showed marked amyloid reduction, suggesting region-specific plasticity that could support motor improvements despite advanced pathology (Diaz-de-Grenu et al., 2014; Li et al., 2014). Specifically, an activity-dependent clearance system has been demonstrated in the motor cortex, possibly mediated by glymphatic or perivascular mechanisms activated during exercise, which could contribute to the selective reduction of β-amyloid observed in this region (He et al., 2017). Overall, these findings suggest that at this advanced stage, exercise may preferentially modulate circuits directly recruited during training, such as the motor cortex, whereas regions with greater structural damage, such as the hippocampus, may show a reduced capacity to respond.

At the peripheral level, no significant changes in the Aβ1-42 relative levels were detected in muscle, this absence of effect may reflect the role of muscle as a peripheral “sink” for amyloid, consistent with reports of exercise modulating peripheral clearance pathways in liver and kidney (Xiang et al., 2015) and with emerging evidence of a bidirectional “muscle-brain axis” in AD (Schlegel et al., 2019). Notably, contracting skeletal muscle releases protective myokines, such as irisin (FNDC5) and other exercise-induced “exerkines” (e.g., cathepsin B, IL-6, lactate), that can cross or signal across the blood–brain barrier and exert neurotrophic, anti-inflammatory, and proteostatic effects (including BDNF upregulation and Aβ clearance support) (Kim et al., 2023; Jodeiri Farshbaf and Alviña, 2021; de Freitas et al., 2020). Future studies should address whether exercise alters muscle Aβ processing or secretion of these protective myokines.

We also observed decreased complex I activity in skeletal muscle and reduced ATPase (complex V) activity in both muscle and brain in sedentary 3xTg-AD mice, whereas complex IV remained essentially unchanged. This pattern suggests a differential tissue vulnerability, with skeletal muscle being susceptible to oxidative stress and peripheral amyloid (Brisendine and Drake, 2023; Monteiro-Cardoso et al., 2015). To our knowledge, this is the first study to evaluate mitochondrial complex activities in skeletal muscle of 3xTg-AD mice and their response to an exercise intervention. After exercise, complex I activity increased in the muscle, complex IV activity increased in the brain, and ATPase activity increased in both tissues. These results align with literature showing that exercise enhances mitochondrial biogenesis and antioxidant capacity via AMPK and PGC-1α signaling (Dos Santos et al., 2024; Liang et al., 2021; Feng et al., 2024), and may counteract ROS-induced damage more effectively in muscle due to direct activation during training (Burtscher et al., 2021).

Although our study focused on amyloid and mitochondria, accumulating evidence indicates that exercise attenuates microglial activation and reduces neuroinflammatory signaling in Alzheimer’s models. In APP/PS1 mice, treadmill training suppresses hippocampal microglia-mediated neuroinflammation and shifts microglial phenotype toward a reparative state; similar anti-inflammatory effects (including lowered pro-inflammatory cytokines) have been reported with exercise paradigms that also modulate the gut-brain axis (Yuan et al., 2022). Likewise, in 3xTg-AD mice, exercise reduces microglial and astrocyte activation and dampens neuroinflammation (Mu et al., 2022). In parallel, exercise promotes synaptic plasticity and elevates neurotrophic support, most notably brain-derived neurotrophic factor (BDNF), in the hippocampus and cortex of AD models, mechanisms linked to improved neuronal survival and function (Sleiman et al., 2016; Choi et al., 2018). These mechanisms may contribute to the motor benefits observed here, even in the absence of cognitive improvement.

Finally, this study found that the correlations between strength and amyloid pathology, body composition, and ATPase activity were stronger than those with locomotor activity or balance parameters, highlighting strength as a particularly informative measure. Muscle strength correlated significantly with amyloid burden and body composition metrics, suggesting that grip strength might function as a meaningful biomarker even in advanced stages and in exercise intervention protocols. Similar correlations have been reported in AD patients, where lower grip strength correlates with reduced brain volume and faster cognitive decline (Meysami et al., 2023; Huang et al., 2022; Boyle et al., 2009), supporting the translational relevance of our observations.

This study has some limitations: (I) it included only male mice and did not assess neuromuscular junctions or direct measures of muscle contractile function; (II) our brain analyses were performed on whole tissue, which may mask regional heterogeneity in mitochondrial dysfunction; (III) future studies should therefore include female subjects, perform region-specific mitochondrial assessments, and investigate early interventions to determine whether exercise can prevent or delay these alterations; (IV) incorporating markers of neuroinflammation, synaptic plasticity, and myokines would also strengthen the mechanistic link between exercise and functional outcomes; (V) The mitochondrial analyses and ELISA assays were performed using a limited number of samples, which represents a methodological limitation. Specifically, the use of whole-tissue homogenates combined with a small sample size may contribute to inter-individual variability and reduce sensitivity for detecting region-specific effects. Accordingly, future studies using larger cohorts and region-specific biochemical approaches will be required to replicate, validate, and consolidate these findings; (VI) electron microscopy analyses were performed as a qualitative ultrastructural assessment based on representative samples (*n* = 1 per group), and therefore did not allow for quantitative evaluation of mitochondrial number or density. This limitation should be considered when interpreting the apparent changes in mitochondrial abundance observed after exercise; (VII) It would also be relevant to assess the correlation between motor and cognitive alterations in response to exercise at earlier stages of the disease. This would help determine whether motor improvements are accompanied by changes in memory and how both domains relate across the course of the disease.

In conclusion, our findings demonstrate that in advanced-stage AD, motor impairments are not only prominent but also accompanied by marked peripheral metabolic and mitochondrial alterations that contribute to sarcopenia and an increased risk of falls. Implementing a mixed voluntary/forced exercise regimen improved motor performance, partially restored mitochondrial function, and selectively reduced amyloid burden in the motor cortex, even in the absence of cognitive benefits. This combined exercise approach mirrors multimodal rehabilitation programs increasingly applied in clinical settings and underscores the potential of tailored physical training to reduce fall risk, counteract sarcopenia, and preserve functional independence. As such, structured exercise interventions emerge as a viable non-pharmacological strategy to mitigate motor deficits and improve the quality of life of patients with late-stage AD.

## Data Availability

The original contributions presented in the study are included in the article/Supplementary material, further inquiries can be directed to the corresponding authors.
