# Supplementary material for: Physical exercise mitigates motor and muscular deficits in the 3xTg-AD model of Alzheimer’s disease
Source: Front Aging Neurosci. 2026 Jan 27;18:1730578. doi: 10.3389/fnagi.2026.1730578 (PMC12886456; doi:10.3389/fnagi.2026.1730578)
Supplement: Supplementary file 1 [file Data_Sheet_1.docx]

# **1 Supplementary Material**

## **1.1 Locomotor activity does not correlate with amyloid pathology or body composition but shows partial association with mitochondrial function**

Locomotor activity (distance traveled) was analyzed in relation to amyloid pathology, muscle morphology, body composition, and ATPase activity in 3xTg-AD mice (Figure 1). No significant correlations were observed between locomotor distance and amyloid burden in the motor cortex (R² = 0.2588, p = 0.1559; Figure 1a) or in the hippocampus (R² = 0.0167, p = 0.7219; Figure 1b). Similarly, no associations were found between locomotor distance and muscle fiber cross-sectional area (R² = 0.0133, p = 0.7507; Figure 1c), muscle tissue percentage (R² = 0.0117, p = 0.6498; Figure 1d), or fat tissue percentage (R² = 0.0117, p = 0.6498; Figure 1e). Interestingly, locomotor distance exhibited a moderate positive trend with both muscle ATPase activity (R² = 0.4079, p = 0.0883; Figure 1f) and brain ATPase activity (R² = 0.4607, p = 0.0642; Figure 1g), suggesting a partial association between locomotor performance and mitochondrial function.


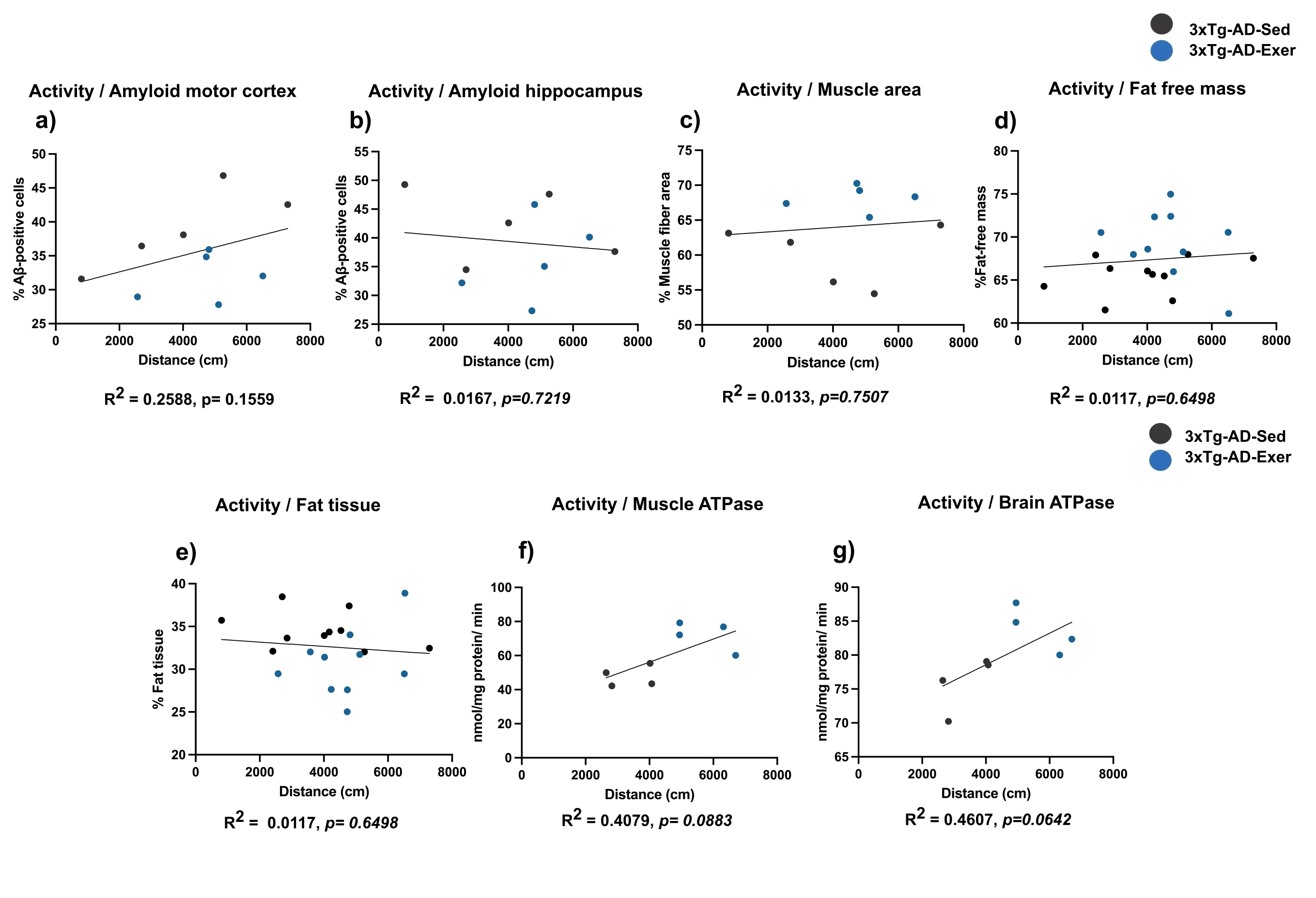


**Figure 1.** Correlation between locomotor activity and amyloid pathology, body composition, and ATPase activity in 3xTg-AD mice. The graphs show the correlation between locomotor distance and amyloid pathology in the motor cortex (a) and hippocampus (b), muscle fiber area (c), muscle tissue percentage (d), fat tissue percentage (e), muscle ATPase activity (f), and brain ATPase activity (g). Pearson’s correlation coefficient and linear regression are indicated in each graph. p < 0.05 was considered a statistically significant difference.

## **1.2 Balance performance does not correlate with amyloid pathology or body composition but shows partial association with mitochondrial function**

Balance performance, assessed as beam walking latency on the 0.5 cm beam, was analyzed in relation to amyloid pathology, muscle morphology, body composition, and ATPase activity in 3xTg-AD mice (Figure 2). No significant correlations were found between balance performance and amyloid burden in the motor cortex (R² = 0.2395, p = 0.1511; Figure 2a) or in the hippocampus (R² = 0.00018, p = 0.9706; Figure 2b). In contrast, a moderate negative correlation was observed between balance latency and muscle fiber cross-sectional area (R² = 0.4046, p = 0.0480; Figure 2c), indicating that animals with smaller fibers exhibited longer crossing times. No significant associations were detected with muscle tissue percentage (R² = 0.1219, p = 0.1313; Figure 2d) or fat tissue percentage (R² = 0.1219, p = 0.1313; Figure 2e). Additionally, no correlation was found between balance latency and muscle ATPase activity (R² = 0.2110, p = 0.2522; Figure 2f). However, a trend toward a negative correlation was observed with brain ATPase activity (R² = 0.4548, p = 0.0666; Figure 2g), suggesting that reduced mitochondrial function in the brain may be linked to poorer balance performance. Overall, balance ability was not significantly associated with amyloid pathology or body composition but showed a partial relationship with central mitochondrial function.


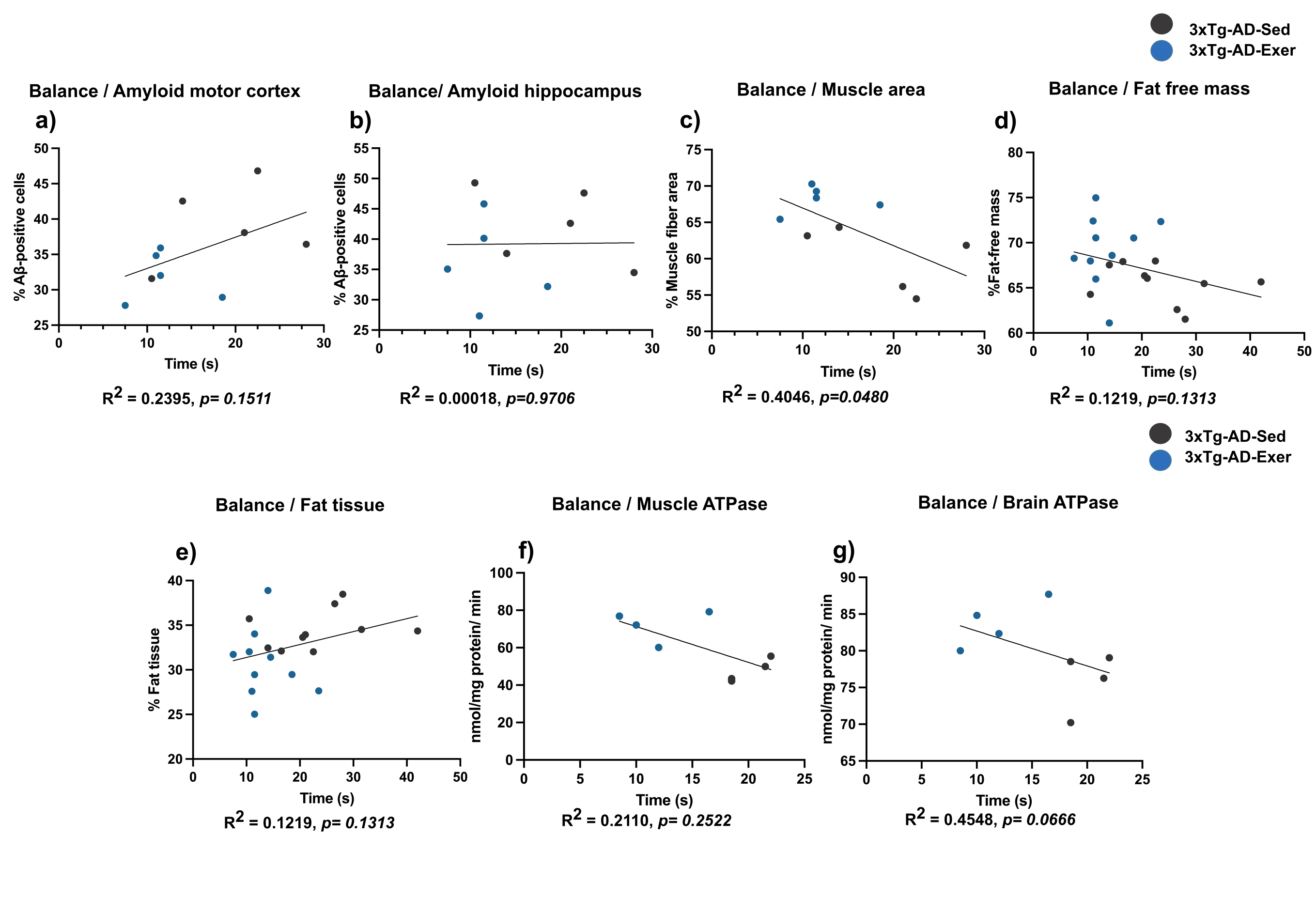


**Figure 2.** Correlation between balance performance and amyloid pathology, body composition, and ATPase activity in 3xTg-AD mice. The graphs show the correlation between beam walking latency and amyloid pathology in the motor cortex (a) and hippocampus (b), muscle fiber area (c), muscle tissue percentage (d), fat tissue percentage (e), muscle ATPase activity (f), and brain ATPase activity (g). Pearson’s correlation coefficient and linear regression are indicated in each graph. p < 0.05 was considered a statistically significant difference.
